# Targeting PP2A in cancer: an underrated option

**DOI:** 10.1186/s13046-025-03560-y

**Published:** 2025-10-27

**Authors:** Fabio Sarais, Finja Krempien, Caroline Koehn, Lara Brewing, Carl Friedrich Classen, Michael Walter, Olia Shokraie

**Affiliations:** 1 Institute of Clinical Chemistry and Laboratory Medicine, Rostock University Medical Center, Rostock, Germany; 2 University Children’s Hospital, Rostock University Medical Center, Rostock, Germany

## Abstract

Protein phosphatase 2A plays a central role in modulating multiple signaling pathways, including PI3K, EGFR, Myc, WNT, JAK/STAT, RAS/MAPK, and NF-κB, which are frequently dysregulated in cancer. Although PP2A is broadly considered a tumor suppressor due to its frequent functional loss in tumors, its biological role is highly context-dependent and varies across cancer types and stages. The activity of the heterotrimeric PP2A complex is tightly controlled by the precise assembly and composition of the scaffolding (A), catalytic (C), and variable regulatory (B) subunits, with the latter exhibiting tissue- and developmental stage-specific patterns, thus allowing PP2A to exert context-dependent effects on signaling networks. In many cancers, PP2A dysregulation arises from mutations in subunit genes, post-translational modifications, or disrupted expression, leading to impaired holoenzyme formation and loss of activity. PP2A dysfunctionality often promotes the aberrant activation of pathways, including mTOR, and the enhanced phosphorylation of own and downstream targets, such as S6 kinase, facilitating tumor growth and progression. In this review, we discuss the mechanisms of PP2A dysregulation in cancer and highlight PP2A restoration as a promising therapeutic approach.

**Graphical Abstract:**

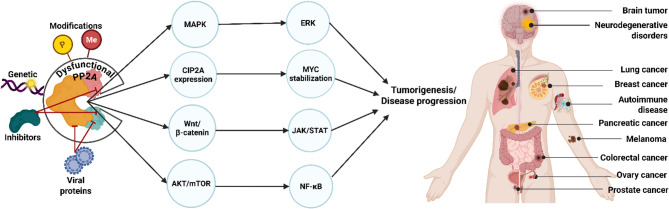

**Supplementary Information:**

The online version contains supplementary material available at 10.1186/s13046-025-03560-y.

## An overview and historical journey of protein phosphatase 2A (PP2A)

PP2A is a crucial member of the tumor suppressor family, and its alteration has been linked to various human malignancies, such as breast [1, 2], oral [3], brain [4], and colorectal cancers (CRC) [5]. This enzyme is a ubiquitously expressed serine/threonine phosphatase localized in different subcellular regions, such as the nucleus, cell membrane, and cytoplasm [6]. The functionality of PP2A is to regulate key signaling pathways, including WNT, mTOR, and MAP kinase, by dephosphorylating over 300 distinct substrates, such as c-MYC, p53, and β-catenin, thereby modulating their activity and stability [7–11]. PP2A displays a broad variety of regulatory subunits and forms diverse holoenzymes involved in essential processes, such as cell proliferation, cell cycle regulation, cell signal transduction, metabolic control, and apoptosis [6]. PP2A constitutes about 1% of all cellular proteins and represents more than 70% of neuronal phosphatases, underscoring its important role in general and in the nervous system in particular [6, 12, 13]. Given its central role in a wide range of cellular pathways, PP2A has earned the title of “master regulator” of the cell cycle, highlighting its importance in maintaining cellular homeostasis [9]. While receptor tyrosine kinases have gained remarkably clinical attention in physiology and cancer therapy [14], suitability as a tumor target is also becoming increasingly apparent for the more abundant serine-/threonine-specific kinases.

**Fig. 1:**
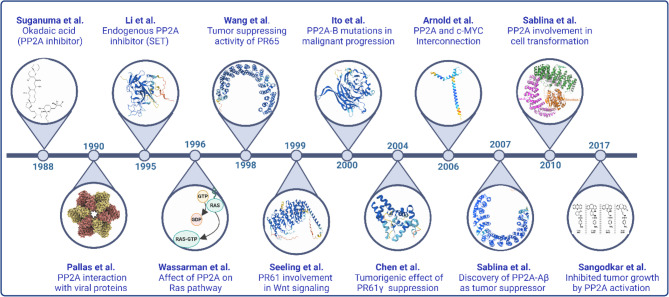
Timeline of key milestones in the discovery of PP2A as a tumor suppressor. This figure highlights the pivotal discoveries that have shaped the current understanding of PP2A’s role in cancer biology. Created using biorender.com

The exploration of PP2A has a rich history, with significant research spanning several decades. Initial studies conducted over the past 30 years have suggested PP2A as a potential tumor-suppressive factor. The key breakthrough was the discovery of okadaic acid, the first potent inhibitor of PP2A [15]. This discovery was followed by Suganuma et al.’s (1988) study, which led to the finding of tumor-promoting activity of okadaic acid in two-step carcinogenesis on mouse skin. This pivotal study provided new insights into the role of PP2A inhibition in tumor progression [16, 17]. The discovery of PP2A as a tumor-suppressive factor set the stage for further investigations into its complex role in cancer, with significant research currently ongoing (Fig. 1). The milestones presented in Fig. 1 highlight the discoveries that have shaped our understanding of PP2A as a tumor suppressor. However, emerging studies have revealed a complex and sometimes contradictory perspective on PP2A’s role in cancer biology. Under certain conditions, PP2A may also facilitate tumorigenesis [18, 19], particularly derived from studies with endogenous inhibitors, such as CIP2A (cancerous inhibitor of PP2A) and SET (SET nuclear proto-oncogene) [4, 20], or non-genomic inhibition [21]. These conflicting findings can likely be attributed to the multitasking nature of PP2A and its subunit composition, exhibiting diverse gene loci and functional roles [22, 23]. The context-dependent activity of PP2A implies that its assembly with different regulatory subunits and interactions with various molecular components and the tumor microenvironment (TME) may determine whether PP2A acts as a tumor suppressor or promotor. Considering the substantial involvement of PP2A in cell cycle regulation, tumor development, and various neurodevelopmental disorders, PP2A has emerged as a promising target for therapeutic approaches in cancer research. However, a comprehensive understanding of PP2A’s multifaceted roles is still limited and vital for developing targeted therapies. Small molecules, such as FTY720 and LB-100, offer the first therapeutic possibilities as modulators of PP2A in cancer treatment [5, 24–26].

### PP2A structure overview

**Fig. 2:**
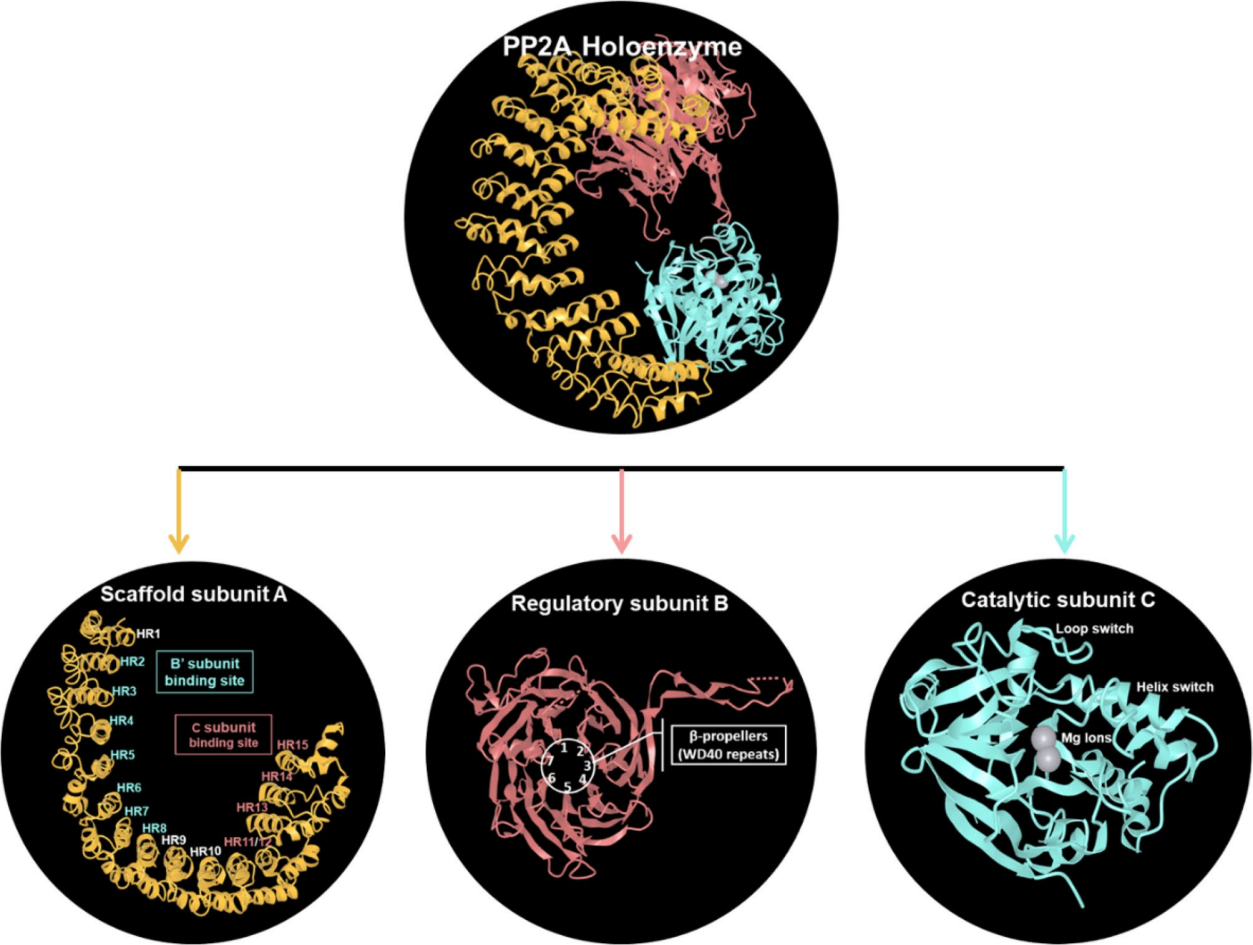
Schematic representation of the PP2A holoenzyme complex and its subunits. The holoenzyme consists of three subunits: scaffolding subunit A (yellow), catalytic subunit C (pink), and regulatory subunit B (pink). The A subunit consists of 15 HEAT repeats with designated binding sites for the regulatory subunit B’ (HR2-HR8) and the C subunit (HR11-HR15), facilitating assembly and spatial organization. The C subunit features structural motifs, loops, and helix switches that contribute to conformational changes essential for enzymatic function. It also coordinates magnesium (Mg²⁺), which is critical for phosphatase activity. The B family shares a typical motif of six β-propeller (WD40) repeats, which is a sequence of 44–60 amino acids (aa), mediating cellular localization, and substrate recognition by providing a platform for protein–protein or protein–DNA interactions [39]. Created in https://www.ncbi.nlm.nih.gov/Structure/icn3d (Access on 23.04.2025)

The active form of PP2A exists as a heterotrimeric holoenzyme composed of three essential subunits. The core enzyme of PP2A (AC dimer) comprises two critical subunits: a 65 kDa scaffolding protein (PP2A-A subunit or PR65), essential for maintaining the structural integrity and assembly of the holoenzyme complex and a 36 kDa catalytic subunit (PP2A-C subunit), which mediates enzymatic activity. This core enzyme combines with a variable regulatory B subunit (PP2A-B) to complete the holoenzyme (Fig. 2) [27, 28]. The A and C subunits are highly conserved in mammals and come in two forms (α and β) [12]. The PP2A-A subunit consists of 15 tandem repeats of a conserved 39-residue sequence known as the Huntington-elongation-A subunit-TOR (HEAT) repeats (HRs) [29]. Structural studies have shown that these 15 h form an elongated, L-shaped structure [30]. The C subunit binds to the conserved ridge of HR 11–15 [31], while the B′ regulatory subunit interacts with the ridge of HR 2–8 (Fig. 2) [27]. PP2A-A and PP2A-C exist in two forms: α and β. The paralogs of PP2A-A (Aα and Aβ) and PP2A-C (Cα and Cβ) share 87% and 98% sequence identity, respectively [29, 32]. These paralogs are encoded by *PPP2R1A* (19q13.41), *PPP2R1B* (11q23.2), *PPP2CA* (5q31.1), and *PPP2CB* (8p12). The Aα-paralog is 10-fold more abundant than the Aβ-paralog [29] and is ubiquitously expressed [33]. It is primarily localized to the cytoplasm across various tissues [34]. The Aβ-paralog is present in higher concentrations in the testis [29, 34], Cα is mainly found in the plasma membrane, and Cβ is located in the cytoplasm and nucleus [35]. More importantly, knockout (KO) studies in mice have revealed that the homozygous deletion of *PPP2R1A* and *PPP2CA* is embryonically lethal [36–38]. This evidence underscores that both α paralogs, the scaffolding (Aα) and the catalytic (Cα) subunits of PP2A, are essential for embryonic development.

Contrary to the A and C subunits, the PP2A-B subunit exists in four subfamilies (Table 1) and is encoded by 15 genes, resulting in at least 26 different transcripts and splice variants [40, 52]. The regulatory subunit is grouped into B (PR55/B55), B′ (PR61/B56), B″(PR48/PR70/PR72/PR130), and B‴ (PR93/PR110/STRN), allowing for over 90 distinct holoenzyme assembly combinations, thus resulting in diverse PP2A functionalities [12, 53]. Each member of the subfamilies displays a unique subcellular localization in the nucleus, cytoplasm, cytoskeleton, mitochondria, Golgi complex, and membranes, as well as tissue-specific expression patterns [54]. For instance, B55γ, B55β, and B56ε are predominantly expressed in the brain [55] and have been associated with brain tumors, neurodegenerative diseases [56, 57], and intellectual disabilities [58]. By contrast, B56α and B56γ are primarily found in cardiac and skeletal muscles, indicating their significance in muscle development and function [40]. The structural diversity underpins their substrate and context specificity, enabling the selective recognition and dephosphorylation of a wide array of target proteins in different physiological settings using a limited and cost-efficient modular system [9].

**Table 1:** List of human regulatory subunits, their chromosomal locations, interactions, structural features, and functions

Subfamily Members, Gene, & Chromosomal Location	Structural Features & Isoforms	Interactions & Functions
B (B55/PR55) Bα: *PPP2R2A*, 8p21.1 Bβ: *PPP2R2B*, 5q31-5q32 Bγ: *PPP2R2C*, 4p16.1 Bδ: *PPP2R2D*, 10q26.3	∎ Seven-bladed β-propellers formed from the WD40 repeat [31] ∎ High sequence similarity [40] ∎ Breakpoint mutations in *PPP2R2B* and *PPP2R2C* [40]	∎ Interaction with HR 1–7 of PP2A-A [40, 41] ∎ Minimal contact with PP2A-C [31] ∎ Providing an acidic surface to Bα-mediating substrate binding [27]
B’ (B56/PR61) B’α: *PPP2R5A*, 1q32.2–q32.3 B’β: *PPP2R5B*, 11q13.1 B’γ: *PPP2R5C*, 14q32 B’δ: *PPP2R5D*, 6p21.1 B’ε: *PPP2R5E*, 14q23.1	∎ Eight Huntington-elongation-A subunit-TOR-like repeats [31, 42] ∎ Three splicing isoforms in B’γ and B’δ [43] ∎ Translational variants of B’ε [43]	∎ Interaction with HR 2–8 of PP2A-A and PP2A-C [31, 42] ∎ Providing an acidic surface to mediate substrate binding at the PP2A-C active site [44, 45]
B’’ (PR48/PR70/PR72/PR130) α: *PPP2R3A*, 3q22.1 β: *PPP2R3B*, Xp22.33, Y11.3 γ (G5PR): *PPP2R3C*, 14q13.2	∎ EF-hand (calcium-binding) motifs to bind the PP2A core enzyme to regulate PP2A functionality [46]	∎ Binding of PR70 to PP2A-A, leading to scaffold extension [47, 48] ∎ C-terminus interaction with PP2A-C, defining the substrate binding site [47, 48]
B’’’ PR93/PR110/striatin(Strn) Strn: *PPP2R6A*, 2p22.2 Strn-3: *PPP2R6B*, 14q13–q21 Strn-4: *PPP2R6C*, 19q13.32	∎ Coiled coil domain ∎ Caveolin-binding domain ∎ Calmodulin-binding domain ∎ WD40 repeat motif [49]	∎ Interaction with PP2A-A and PP2A-C ∎ Mediating dephosphorylation in cellular processes ∎ Interaction with kinases to form large signaling complexes [50, 51]

The mechanism of how the individual regulatory B subunits identify the AC dimer for assembling into the holoenzyme remains partially understood [33, 59, 60]. Limited insights have been gained from the B’ subunits, which require an intact A subunit for binding, as deduced from in vitro experiments and mouse models. The structural diversity among the B subunit subfamilies hints at various potential recognition mechanisms. The application of mass spectrometry and high-resolution imaging techniques, including crystal and cryo-electron microscopy, has uncovered interactions between the B subunit and the core AC dimer. Notably, much of the research has focused on the B55 and B56 subfamilies of the B subunit [61, 62]. Initial studies have pointed to the reversible methylation of a specific residue (L309) at the C-terminal tail of the catalytic subunit [63–65]. This modification appears to be critical in modulating the binding affinity of the regulatory subunit, contributing to the formation of distinct heterotrimeric complexes. Interestingly, the sensitivity to carboxymethylation varies among different B subunits, with B55, B56α, and B56ε exhibiting higher sensitivity than B56γ and B56δ. By contrast, the PR72 and Strn subfamilies showed independence from the carboxymethylation of the AC dimer [66]. Furthermore, several studies have reported the identification of short linear motifs (SLiMs), such as LxxIxE [61] and LSPIxE [62]. These motifs play a pivotal role in substrate targeting and facilitating specific protein–protein interactions. For instance, the LxxIxE motif in substrates binds to a conserved groove in the B56 subunits, with phosphorylation enhancing binding affinity [61]. Although SLiMs are not centrally associated with the initial holoenzyme assembly, substrate recognition by specific B subunit members may regulate the dynamic aspects of holoenzyme assembly, stability, or subunit exchange, indicating a degree of flexibility and responsiveness within the assembly process despite structural constraints. Padi et al.’s cryo-electron microscopy study showed that the B55 subunit employs a unique non-SLiM interface to engage with substrates such as p107 and Eya3. This interaction relies on a distinct peptide-binding groove unique to B55, which is regulated by phosphorylation [67]. Such findings underscore that the recognition mechanisms of B subunits are specific to their family and structurally defined, shaping a modular and adaptable framework for the assembly of the PP2A holoenzyme.

### Common mechanisms of PP2A regulation and dysregulation

**Fig. 3:**
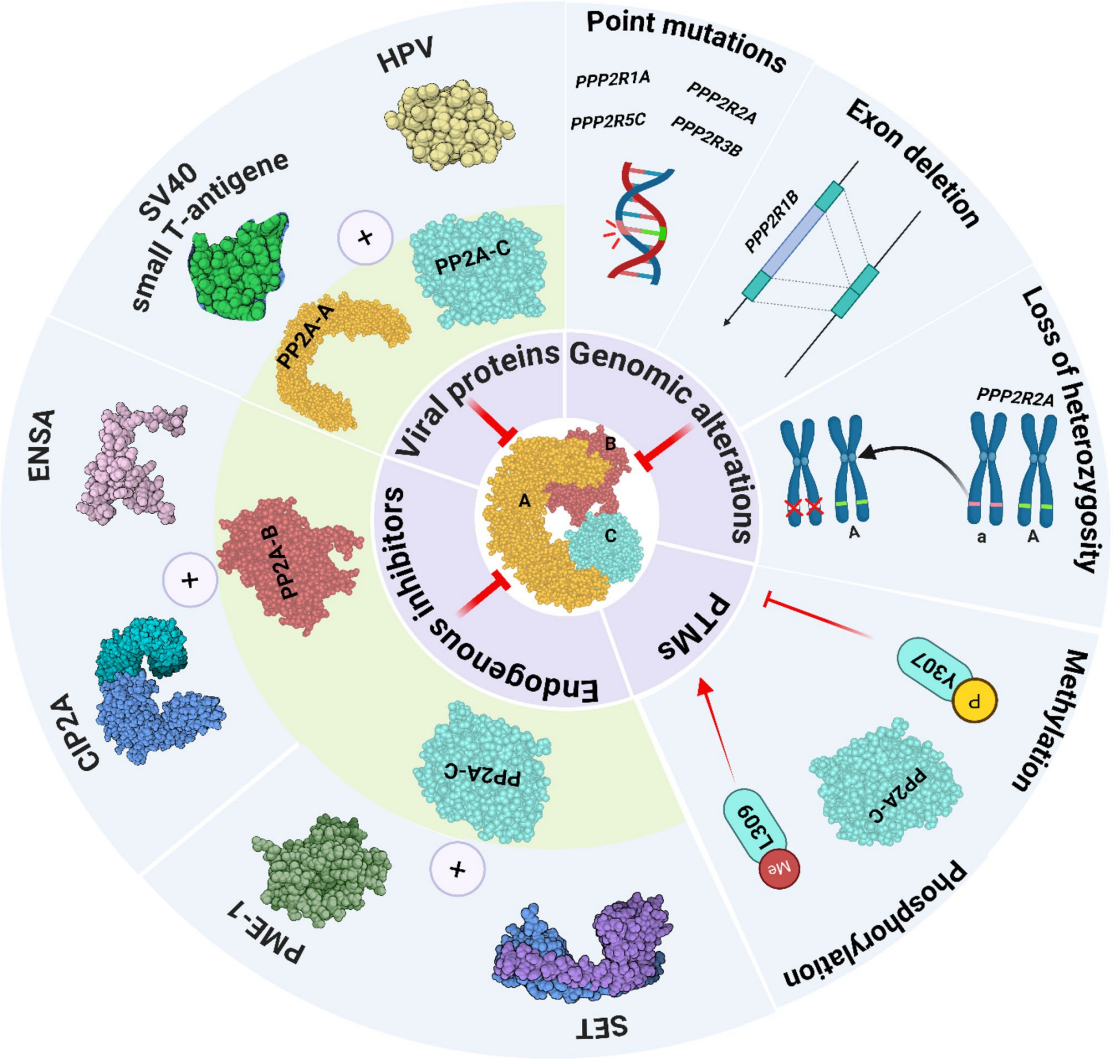
Common mechanisms contributing to PP2A dysregulation. This figure depicts the key regulatory factors that influence the structural integrity and enzymatic activity of PP2A. Genomic alterations, PTMs, endogenous inhibitors, and viral proteins can all affect the subunit composition, assembly, and function of the PP2A holoenzyme. Disruption of the PP2A assembly may result in reduced, insufficient, or complete loss of phosphatase activity, thereby perturbing downstream signaling pathways and ultimately leading to diseases. Created using biorender.com

The regulation of PP2A is controlled through multiple mechanisms to maintain cellular homeostasis and prevent uncontrolled proliferation [68, 69]. Mis- and dysregulation due to aberrant genes, post-translational modifications (PTMs), endogenous inhibitors, or disturbed protein interactions (Table 2) lead to impaired, incomplete, or dysfunctional PP2A activity, contributing to chronic diseases and carcinogenesis (Fig. 3) [101–103]. Among these, genetic alterations in the PP2A subunit genes, namely point mutations, deletions, and loss of heterozygosity (LOH), play a central role in disrupting PP2A functions and contribute to tumorigenesis [104, 105]. For example, *PPP2R1A* mutations have been observed in several cancer entities, including uterine serous carcinoma (USC) and uterine carcinosarcoma (UCS) [104, 105]. Here, PP2A plays a critical role in modulating paracrine signaling pathways, particularly through its regulation of insulin-like growth factor binding protein 2 [104].

**Table 2:** Common mechanisms of PP2A regulation vs. dysregulation

Mechanism	Involved in Regulation	Consequences of Dysregulation
Genetics	∎ Holoenzyme assembly ∎ Cell cycle control [70–72] ∎ Cell death [73, 74] ∎ Cell differentiation [74] ∎ Embryonic, organ, and/or tissue development [75]	Mutations in: ∎ *PPP2R1A* impaired holoenzyme formation by disrupting the binding of the regulatory B’ subunits in lung, breast, and endometrial carcinomas [76] ∎ *PPP2R2A* reduced tumor-suppressive functions in prostate [77], ovarian, and breast cancer patients [1] ∎ *PPP2R5C* reduced tumor-suppressive functions in lung cancer [78] Upregulation of *PPP2R2A* (PR55α) was observed in pancreatic ductal adenocarcinoma (PDAC) independent of mutation [79] Downregulation of *PPP2R3B*, linked to X chromosome inactivation, was associated with tumorigenesis in females with breast, ovarian, and melanoma tumors [80]
		Loss of heterozygosity in: ∎ *PPP2R1B* led to the generation of a dysfunctional protein incapable of binding to the C subunit [81] ∎ *PPP2R2A* resulted in reduced mRNA expression in lung cancer [82, 83] ∎ Exon deletion in *PPP2R1B* was linked to the emergence of loss-of-function (LOF) variants in breast [84] and lung cancer [81]
Post-Translational Modifications	∎ Holoenzyme assembly - Methylation of PP2A-C at L309 by LCMT-1 increases the AC dimer binding affinity to certain B55/B56 subunits [85–87] - Phosphorylation of PP2A-C at T304 by CDK1-cyclin B disrupts holoenzyme formation by inhibiting the binding of B55 [88] - Ubiquitination of PP2A-C promotes its degradation; the Alpha4–MID1 complex inhibits this process by preventing polyubiquitination [89, 90] ∎ Signal transduction [21]	Aberrant modifications: ∎ Phosphorylation of PP2A-C at Y307 by Src family kinase-dependent signaling inhibits its catalytic activity, prevents methylation, and thus inhibits the binding of B55 [91]. This is associated with a higher lymph node stage and shorter survival times in oral squamous cell carcinoma [3]
Protein Interactions	A proper network results in a timely and spatially localized (balanced) PP2A activity in key targets.	Endogenous PP2A inhibitors ∎ The binding of the SET protein to the C subunit completely inhibits its activity, functioning as an oncogene [92] ∎ PP2A methylesterase 1 (PME-1) directly binds to the active site of the C subunit, demethylating L309 and thereby inactivating PP2A [93, 94] ∎ CIP2A interacts with B56α and B56γ, preventing their assembly into the holoenzyme [95, 96] ∎ α-endosulfine interacts with B55δ, impeding its activity [97]
Viral Infections		∎ Small t-antigen of simian virus 40 (SV40) interacts with the A subunit, altering PP2A activity [98, 99] and thus affecting genes involved in cell transformation [99] ∎ The binding of human papilloma virus E7 to the C subunit prevents holoenzyme formation, thereby promoting cell proliferation [100]

Another level of regulation occurs after transcription, particularly in PTMs. This includes the reversible methylation (Me) of leucine (L) and the phosphorylation (P) of tyrosine (Y) residues at the C-terminal end of the catalytic subunit. Methylation regulates physiological PP2A activity, whereas phosphorylation may inhibit enzyme activity by preventing the interaction of the C subunit with B55/B56 complexes, thereby reducing the phosphatase activity [65].

Another layer of PP2A regulation involves proper interactions within other proteins, ensuring balanced phosphatase activity. In this context, several studies have identified PP2A endogenous inhibitors (Table 2) that interfere with enzyme function. Crespo et al. (2024) reported that interactions between PP2A, its regulators, such as the immediate early response family genes, and PP2A target proteins may influence tumorigenesis in aggressive prostate cancer, in which these regulators are often upregulated [106].

### Comparative PP2A dysregulation across cancer types

Dysregulation of PP2A is a key driver of cancer development, most often arising from copy number variations (CNV), imbalances in regulatory proteins (SET and CIP2A), or PTMs rather than recurrent hotspot mutations. These changes typically involve altered expressions of PP2A regulators and specific regulatory B subunits, while the catalytic (C) and scaffolding (A) subunits are less frequently affected [65, 107]. For instance, in the glia cells of brain tumors, glioblastoma (GBM) downregulation or loss of the regulatory subunit *PPP2R2C* has been reported [10]. *PPP2R2C*/B55γ is highly expressed in healthy brain tissue, particularly in astrocytes. Notably, the B55γ level was found to correlate inversely with the malignancy stage. The restoration of *PPP2R2C* expression in glioma cells inhibited colony formation and suppressed cancer cell proliferation in both in vitro and in vivo models [10]. Conversely, *PPP2R2C* overexpression led to decreased S6 kinase (S6K) phosphorylation, a downstream target of the mTOR pathway, suggesting S6K as a key substrate of B55γ [10, 108]. Through the dephosphorylation of S6K and possibly by indirectly downregulating Akt phosphorylation, B55γ seems to inhibit the mTOR pathway, thereby suppressing protein synthesis and preventing or delaying uncontrolled and abnormal cell growth [109]. These findings highlight the tumor-suppressive role of *PPP2R2C*/B55γ in glioma, potentially pushing GBM cells into a therapeutic “dead-end,” limiting their ability to evade treatment. PP2A dysregulation has also been documented in neuroblastoma and metastatic CRC, primarily due to the elevated levels of endogenous PP2A inhibitors CIP2A [110] and SET [101]. More importantly, *CIP2A* overexpression has been detected in a broad range of malignancies, including hepatocellular carcinoma (HCC) [111] and bladder carcinoma, in which its expression correlates with disease progression [112]. In polycythemia vera and other myeloproliferative neoplasms, PP2A’s tumor-suppressive function is inhibited by phosphorylated SET, a process mediated through JAK-STAT and PI3K-PKC signaling [113]. In addition, downregulation of the B56α regulatory subunit has been observed in melanoma. Lower activity influences the stability and accumulation of the c-MYC oncoprotein, enhancing its activity and suppressing oncogene-induced senescence [114]. However, in breast cancer, PP2A dysfunction is linked to the reduced expression of both scaffold (PP2A-Aα and PP2A-Bα) and regulatory (PP2A-B′α) subunits. One mechanism contributing to this dysregulation is a mutation in the scaffold subunit PP2A-Aα, which disrupts the interaction between the scaffold and regulatory subunits, leading to the hyper-proliferation of breast cells [1]. It should be noted that among the PP2A subunits, the paralogs of the A subunit exhibit the highest frequency of mutations across a variety of cancer types. These mutations are mainly recurrent, pathogenic, and frequently observed at the interface of the binding sites between the A and B subunits [107]. Table 3 summarizes most of the characterized mutations within PP2A subunits, highlighting the variants classified as oncogenic/likely oncogenic, including those associated with hotspots and other mutations with biological effects linked to cancer progression.

**Table 3:** List of potential mutational hotspots in PP2A subunits in various cancer entities

Subunit	Cancer Context	Protein Change	Functional Impact
*PPP2R1A *(Aα)	Breast carcinoma [84, 115]	E64G del171–589 ^90,^[115]	Mutant proteins are defective in binding to the B subunits, the C subunit, or both ^90,[^115].
	Melanoma ^90,^[115]	R418W ^90,^[115]	
	Lung carcinoma ^90,^[115]	E64D ^90,^[115]	
	Breast ductal carcinoma [116] Colon adenocarcinoma [116] Lung adenocarcinoma [116] Ovarian clear-cell carcinoma [117]	R183W [116, 117]	These hotspot LOF mutations in HR5 and HR7 disrupt interactions with PP2A-C [118], B55, and B56 subunits [116, 118, 119], impairing the PP2A holoenzyme assembly [119].
	Head and neck carcinoma (HNC) Uveal melanoma (TCGA)	R183Q	
	Mucinous carcinoma (TCGA)	S256Y	
	Serous ovarian carcinoma (TCGA)	P179R, R183W	
	Stomach adenocarcinoma (TCGA)	R182W, R183Q	
	Uterine endometrial Carcinoma (serous carcinoma and carcinosarcoma) [76]	P179L/R/T, R182W, R183W/Q, S256F/Y, W257C/G/L/S/, R258H, R418W [76]	
*PPP2R1B* (Aβ)	Breast cancer [120] Lung cancer [120]	G90D [120]	Biochemical defect in the Aβ protein disrupted the interaction with the B56γ subunit [120].
	Colon adenocarcinoma [121]	I338F, R430S, S457F, A469T, W489C (TCGA)	Deletions or point mutations within aa 412–601 impeded the interaction between PP2A-Aβ and PP2A-C [121].
	Breast carcinoma (LOF variants) [122]	E6*, V115fs, R194*, R233C [122]	Truncated Aβ products are unstable and fail to bind PP2A-C, leading to loss of phosphatase activity [122].
*PPP2CA* (Cα) *PPP2CB* (Cβ)	Lack of canonical cancer hotspots or known oncogenic mutations (TCGA).		
*PPP2R2A* (Bα)	Acute myeloid leukemia (AML) [123]	E83*, G29* (LOF) [123], X278_splice (TCGA)	LOF mutations suppressed B55α expression, allowing aberrant Akt activation [123].
	Colon adenocarcinoma (TCGA)	E41*, N106Kfs*11, S282Pfs*10	Truncated mutant proteins often lack residues essential for binding to the A and C subunits [123].
	GBM multiform (TCGA)	E70*	
	Lung carcinoma (TCGA)	E83*, E91*, R158Kfs*8, R298*, X153_splice	
	Rectal adenocarcinoma (TCGA)	E41*, S282Pfs*10	
	Serous ovarian cancer (SOC) (TCGA)	X268/323_splice	
	Uterine endometrial carcinoma (TCGA)	E41*, G46*, E271*, R298*, W424*, X268/355_splice	
*PPP2R2B* (Bβ)	Lack of canonical cancer hotspots or known oncogenic mutations (TCGA). Promoter hypermethylation in CRC resulted in loss of tumor suppressor activity [124].		
*PPP2R2C* (Bγ) *PPP2R2D *(Bδ)	Lack of canonical cancer hotspots or known oncogenic mutations (TCGA).		
*PPP2R5A (* B’α) *PPP2R5B* (B’β) *PPP2R5D (*B’δ) PPP2R5E (B’ε)	Lack of canonical cancer hotspots or known oncogenic mutations (TCGA). Cancer relevance to these genes tends to arise from dysregulated expression, CNV, or indirect mechanisms that alter holoenzyme composition or targeting [114, 125–129]		
*PPP2R5C* (B’γ)	Lung adenocarcinoma [23]	F395C [23]	The B56γ-p53 interaction was disrupted, leading to the loss of tumor suppressive function [23].
*PPP2R3A* (B’’α)	Childhood acute lymphoblastic leukemia [130]	DNA methylation [130]	Hypermethylation of the *PPP2R3A* promoter led to its inactivation, blocking PP2A holoenzyme assembly [130].
*PPP2R3B* (B’’β)	Lack of a validated hotspot residue with clear driver evidence (TCGA). Germline *PPP2R3B* duplications in melanocytic nevi and melanoma promoted proliferation and reduced migration [131].		
*PPP2R3C* (B’’γ)			
*PPP2R6A* (Strn)	Mesothelioma [132] Papillary thyroid cancer (TCGA) [133] Papillary renal cell carcinoma (TCGA)	STRN-ALK fusion [133]	STRN-driven ALK activation promoted cell proliferation independently of thyroid stimulating hormone, caused oncogenic transformation and induced tumors in mice [133].
*PPP2R6B* (Strn-3) *PPP2R6C* (Strn-4)	Lack of canonical cancer hotspots or known oncogenic mutations (TCGA).		

Guidelines for the nomenclature- del: deletion; fs: frameshift; splice: RNA splicing/splice variant; *: stop codon

*C* Cysteine, *D* Aspartic Acid), *E* Glutamic Acid, *F* Phenylalanine, *G* Glycine, *H* Histidine, *I* Isoleucine, *K* Lysine, *L* Leucine, *N* Asparagine, *P* Proline, *Q* Glutamine, *R* Arginine, *S* Serine, *T* Threonine, *V* Valine, *W* Tryptophan, *Y* Tyrosine

The other paralog of the A subunit (PP2A-Aβ) also shows dysregulation, with exons 2–3, 3, and 9 skipping reported in B-cell chronic lymphocytic leukemia (CLL). These splicing events generate alternative variants that reduce PP2A activity. Interestingly, skipping exons 3 and 2–3 was also detected in control cases, suggesting that a dose effect or a gatekeeper function may exist to trigger pathological effects. The loss of exons 2–3 or 9 disrupts the B subunit binding domain, impeding holoenzyme formation while allowing the dimerization of A and C subunits. Furthermore, premature translation termination might occur due to the exon 3 deletion (del), leading to truncated proteins that lack essential interaction domains [134]. The LOH of *PPP2R2A* in non-small cell lung cancer (NSCLC) [83] and *PPP2R1A* mutations in USC and UCS [104] represent additional genetic mechanisms contributing to PP2A dysregulation. One notable mutation is P179R, in which arginine (R) is replaced by proline (P) at residue 179. This missense mutation alters the conformation of the A subunit, reducing its binding affinity for the C subunit and disrupting overall enzyme activity [18]. PP2A also plays a role in the tumor progression of gastric cancer, in which *STRN3* inactivates mammalian Hippo kinases MST 1/2 through dephosphorylation [135]. In addition, the dysregulation of PP2A in acute myeloid leukemia (AML) was associated with reduced B55α transcript levels compared with normal CD34⁺ cells [102].

Extending the observations from in vitro studies, the findings from mouse models suggest that overall reduced PP2A activity and impairments of the structural and regulatory B subunits have a tumor-promoting effect (Table 4).

**Table 4:** Mouse model studies of PP2A subunit mutations across cancer types

PP2A Subunit	Mouse Model/Modified Protein	Non-cancerous Effect	Cancerous Effect	Proposed Mechanism
*PPP2R1A* (U/ES/S)	Aα KO [36, 136]	Lethal		
	Aα KI E64D/+ Aα KI Δ5–6/+ Aα KI Δ5–6/E64D [36, 137]		50%↑ lung cancer (BP)	Defective binding to B’ subunit(s)
	Aα KI E64D plus dnp53 [36]		Unchanged lung cancer	p53 required for tumor suppression
	Aα KI E64D/+ [137] plus/wo K-Ras^G12D^		Mice death 3.5 weeks earlier	RAS-MAPK is a target of PP2A tumor suppressor effect
	Aα KI D 5–6/+ plus/wo K-Ras^G12D^		Mice death 3 weeks earlier	
	Aα KI Δ5–6/E64D plus/wo K-Ras^G12D^		Mice death 7 weeks earlier	
	Aα cKO (all tissues) [36]	Weight loss and death on day 6		
	Aα cKO (O) [138]	↑Aneuploidy Subfertility Chromosome segregation		↑ p-Rec8-cohesin
	Aα cKO (T-cells) [139]	↑Autoimmunity		↑Effector immune response ↑mTORC1
	Aα cKO (B-cells) [140]	↓B cell survival		Impaired redirection of glycolysis to PPP
	Aα transgene (M) [141]	Cardiomyopathy		
*PPP2R1B* (U/ES/S)	Aβ [142]	Male infertility		Meiotic arrest in spermatocytes
*PPP2CA* (U/ES/S/E)	Cα KO [38]	Lethal (embryo)		No mesoderm formation
	Cα cKO (G) [37]	Lethal		
	Cα cKO (O) [143]	Female infertility		Chromosomal misalignment Impaired AurK B/C inhibition
	Cα cKO (G) [144]	Male infertility		
	Cα cKO (H.C.) [145]	Lethal ↓Fetal liver erythropoiesis		↑Apoptosis ↓p-STAT5 Impaired Bclx signaling
	Cα cKO (Ma) [146]	↑Antiviral response		↑Type I IFN signaling
	Cα cKO (H) [147]	↑Glycogen storage		↑Insulin sensitivity ↑Akt
	Cα cKO (H) [148]	↓Liver injury (CCl4^−^)		↓TGF-ß1/SMAD signaling
	Cα cKO (CNS) [149]	Microcephaly ↓Plasticity		↑Hippo-p73 signaling ↑Apoptosis
	Cα cKO (C) [150]	Hypertrophy ↑Glycolysis ↑ß-oxidation ↓ Fatty acid transport		↑p-PLN
	Cα cKO (E) [151]	Smaller animals		↓Akt and Wnt signaling
	Cα transgene (CNS) [152, 153]	Tau aggregation		↑p-Tau ↑ERK and JNK
	Cα transgene (Os) [154]	Larger mice ↑Bone formation ↑Adipogenesis		↑PPARγ ↑CBPα ↑Adipogenesis
	Cα transgene (CNS) [155, 156]	Tangles ↓CNS development		↑p-Tau ↑p-Vimentin ↑Akt/p-Akt
	Cα ↑ transgene (C) [157]	Hypertrophy		↓PLN and eEF2 phosphorylation
	Cα ↑ transgene (C) [158]	Cardiomyopathy		↓Akt/GSK3β/β-catenin
	Cα ↑ transgene (T-cells) [159, 160]	Glomerulonephritis		↑IL17 Impaired chromatin remodeling
*PPP2CB* (U/ES/S/E)	Cβ cKO (G) [37] Cβ cKO (O) [143]	Viable Infertility		
*PPP2R2A* (ES/S/E)	B55α ↓ (H.M.) [161] B55α ↓ (H.M.) plus IR-KO [161]	↓Insulin sensitivity Type 2 diabetes		↓Akt/p-Akt signaling ↓Akt/p-Akt signaling
*PPP2R2B*	B55β cKO (T-cells) [162]	Impaired clonal contraction of CD8^+^ T-cells		↑p-Akt, thereby preventing FoxO and Hrk-dependent apoptosis
*PPP2R2C* (U/ES/S/E)	N.A.			
*PPP2R2D* (ES/S)	N.A.			
*PPP2R5A* (U/S/ES)	B56α cKO [163]	↑Arrhythmia		↑PP2A activity (lack of autoinhibition)
	B56α ↓ (H.M.) [164]		Spontaneous skin lesions ↑Inflammation	Tumor initiation via p-MYC Hyperproliferation Impaired stem cell homeostasis
	B56α ↑ transgene [165]	↑(Basal) Inotropy ↑Heart rate ↓(Iso)Contractility		↑PP2A activity ↓p-cTnI ↓p-MyBP-C ↓p-PLN
*PPP2R5B* (U/ES/S)	B56β KO	N.A.	N.A.	N.A.
*PPP2R5C* (U/ES/S)	B56γ KO [75]	Neonatal death ↓Ventricular tissue		↑Apoptosis
	B56γ ↓ (H) [166]	↑Glucose tolerance ↑Lipogenesis		↑AMP kinase ↑HIF1a ↑SREBP1
	B56γ ↑ transgene (L) [167]	Neonatal death		Lung differentiation defect
*PPPR5D*	B56δ KO [168, 169]	Homozygotes: lethal Heterozygotes: Abnormal behavior and coordination; Tauopathy [168]	Spontaneous hematological malignancies and HCC	p-Tau at pathological sites ↑GSK3β, ↓CDK5, ↓p35 [168] ↑p-MYC oncogenicity ↑p-GSK3β [169]
*PPPR3A* (U/ES/S/E)	N.A.			
*PPPR3B*	N.A.			
*PPPR3C* (E/S/ES)	B‘‘γ cKO (B-cells) [170]	↓B-cell development		↑Apoptosis ↑c-Jun ↑Bim
*PPPR3C* (E/S/ES)	B‘‘γ cKO (T-cells) [171]	↓T-cell development		↑Apoptosis ↑JNK/FasL ↑Caspase3
*PPPR3D* (U)	B‘‘γ ↑ transgene [172]	↓Antibody production ↑Autoimmunity		↓Affinity maturation of Ag-specific B-cells
*PPPR6A* (U/ES/S/E)	STRN KO [173, 174]	Homozygotes: lethal Heterozygotes: ↑Blood pressure;↓ relaxation		↓p-Akt/Akt-ratio NO-cGMP signaling disturbed
*PPPR6B*	N.A.			
*PPPR6C*	N.A.			

*AurK B/C* aurora kinase B/C, *C* cardiomyocyte, *CCl4-* carbon tetrachloride, *cKO* conditional Knockout, *cTnI* cardiac troponin inhibitor, *dnp* dominant negative mutant, *E* epidermal, *eEF2* eukaryotic elongation factor 2, *FoxO* forkhead box O, *G* germline, *H* hepatocyte, *H.C*. Hematopoietic cells, *H.M.* hypomorphic mutation, *Hrk* Harikiri, *I.R*. insulin receptor, *Iso* isoproterenol, *L* lung, *M* muscle, *Ma* macrophage, *MBP* myelin basic protein, *MyBP-C* myosin-binding protein C, *N.A.* not available, *O* oocytes, *Os* osteoblast, *PPP* pentose phosphate pathway, *PLN* phospholamban, *E/ES/S/O/U* available as cryopreserved embryo, ES cell line, sperm, ovaries, or as unspecified material, according to the International Mouse Strain Resource (www.findmice.com)

Walter et al. developed several knock-in models with mutations of the Aα structural subunit typically observed in cancer [36]. Consistent with previous in vitro studies in which Aα mutations fully disrupt B subunit binding [115], Aα knock-in mice (E64D/+) showed a maximum tumorigenic effect similar to mice with deletions of exons 5 and 6 in the Aα allele, with an additional halving of the entire holoenzyme [115]. In particular, the E64D/+ heterozygote mutation and heterozygote deletions of exons 5 and 6 (del5-6/+), as well as the double mutant del5-6/E64D, showed an approximately 50% increased lung cancer rate after benzopyrene exposure compared with wild-type mice. This increase could be reversed by the simultaneous expression of a dominant-negative *TP53* allele, suggesting that PP2A exerts its tumor-inhibiting effect via the p53 signaling pathways within the PI3K/AKT axis. Apparently the E64D/+ mutation prevents the proper formation of the B’ holoenzyme, leading to the loss of tumor suppressor activity and undermining the tumor-suppressive maximum [115]. Furthermore, Aα-KI models were crossed with transgenic mice ubiquitously expressing the mutated KRAS^G12D^ allele [175]. This led to a significantly reduced median survival time in lung adenocarcinoma compared with KRAS^G12D^ mice with wild-type Aα [137], suggesting RAS/MAPK as a target of the PP2A tumor suppressor effect. PP2A should normally dephosphorylate and inactivate MEK and extracellular signal-regulated kinase (ERK) and/or could increase p53 levels via the ARF-MDM2-p53 mechanism linked to the RAS/MAPK pathway.

The tumor suppressor effect of B’ subunits is further demonstrated through direct manipulation. In B56α KO mice, DMBA/TPA treatment resulted in an accelerated appearance of skin papillomas. The loss of B56α did not affect the progression to squamous cell carcinoma, suggesting that B56α primarily inhibits tumor initiation [164]. Skin cells lacking B56α can possibly not adequately control c-MYC activity during oncogenic stress, thereby promoting tumor initiation [164]. Particularly striking in the B56δ KO model was the high incidence of malignant lymphomas (predominantly non-Hodgkin B-cell lymphomas) in aging animals. Furthermore, up to 60% of the oldest mice developed HCC. Transcriptome analyses identified enhanced c-MYC activity in HCCs, supported by increased c-MYC phosphorylation at the S62 residue and enhanced the expression of CIP2A (a MYC target) in all analyzed tumors. A previously described negative feedback mechanism between B56δ and c-MYC [176] may explain these findings. Similar results were observed in heterozygous phosphotyrosyl phosphatase activator (*PTPA*) KO mice. *PTPA en*codes for an activator of PP2A, indicating that the overall activity of PP2A B’56γ/ε (and presumably other B56 isoforms) was reduced in favor of tumorigenesis by increasing susceptibility to DMBA/TPA-induced skin papillomas [177]. Furthermore, the reduction of certain B’56 subunits increased likelihood of spontaneous tumors, for example, in the complete KO of the B’56δ subunit [169] or in the homozygous and heterozygous hypomorphic alleles of *PTPA* [177].

In summary, the PP2A cancer models mentioned above illustrate the tumor-inhibiting role of PP2A B’ complexes, especially the isoforms B56α and B56δ, and how they prevent tumor initiation in a context- and tissue-specific manner. These findings are consistent with data from human mutated tumors. The fact that no increased cancer rate has been described in other regulatory subunits in mouse models, including some for which tumor associations have been proposed in humans, does not mean that they do not exist in mice. In most cases, very specific questions, such as involvement in neurodegenerative diseases, were the focus. It is striking that typical tumor suppressor signaling pathways were often altered in these studies, which also fits with the suspected overall functions of these subunits. These include Akt, MYC, and MAPK, which are commonly hyperactivated in cancers. Moreover, some subunits have not yet been manipulated in mice, although some embryos are already commercially available. Thus, it can be expected that further tumor-suppressive mechanisms will be identified in mouse models.

## PP2A as a therapeutic target

### Clinical relevance and therapeutic implications

**Fig. 4:**
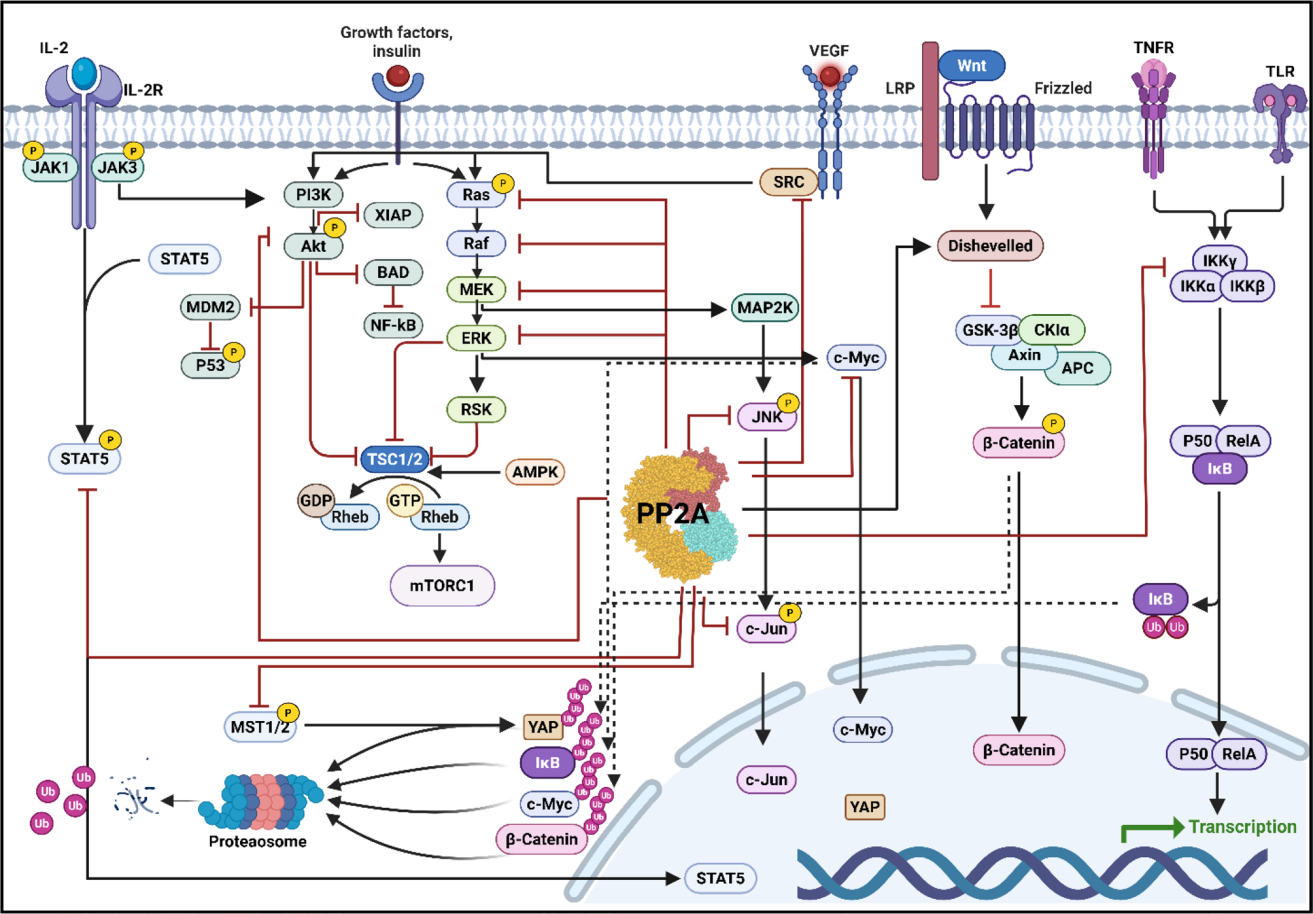
Signaling networks of PP2A. PP2A is a key regulator of cellular processes, including protein synthesis, cell cycle progression, proliferation, differentiation, and apoptosis. It modulates β-catenin stability in the Wnt pathway, controlling cell proliferation. PP2A balances the signals, determining the cell fate for proliferation or differentiation by fine-tuning phosphorylation. It also maintains proper cell cycle progression by regulating G1/S cyclins and ensuring cell survival by modulating mitogenic signals. Loss of PP2A function leads to apoptosis resistance by hyperactivating survival pathways, particularly the MAPK/ERK and PI3K/AKT cascades. Normally, PP2A counterbalances these pathways to maintain cellular homeostasis and prevent aberrant cell survival. As a central node in multiple signaling networks, PP2A dysfunction contributes to pathological conditions, such as cancer and neurodegeneration. Created using biorender.com

PP2A exhibits a context-dependent duality in cancer biology, functioning as both a tumor suppressor through its phosphatase-mediated inactivation of growth-promoting kinases (e.g., PI3K/AKT and MAPK/ERK) and MYC-driven transcription and as a potential tumor promoter when its regulatory subunits are hijacked to stabilize survival pathways or when specific holoenzyme complexes paradoxically enhance oncogenic signaling networks (Fig. 4) [104, 178, 179].

The tumor suppressor activity of PP2A is exerted predominantly through its ability to negatively regulate kinase-driven signaling pathways that may drive uncontrolled proliferation and survival [180]. Central to these pathways is the reversible phosphorylation of key signaling molecules, a molecular switch that determines their activation status and functional output. The disruption of this balance, particularly through the dominance of hyperactive kinases, profoundly distorts growth regulatory networks [181]. Indeed, many proto-oncogenes encode protein kinases [182], and several canonical oncogenic signaling cascades are subject to tight regulation by PP2A. PP2A dephosphorylates Akt at critical residues, thereby limiting its kinase activity [183] and functioning as an antagonist of ERK [184]. This process effectively curbs uncontrolled proliferation, often driven by excessive mitogenic signaling. PP2A also exerts a negative regulatory effect on the Wnt/β-catenin pathway, leading to the degradation of β-catenin and, consequently, the limitation of stemness-associated target genes [185]. Furthermore, PP2A regulates c-MYC by dephosphorylating key residues, with PP2A-B55α targeting S62 to stabilize c-MYC and PP2A-B56α targeting T58 to promote its degradation, thus regulating tumor growth [186–188].

The reconciliation of PP2A dual roles is attributable to its mechanistic diversity, which encompasses subunit composition, PTMs, and interactions with regulatory proteins. Therefore, the holoenzyme assembly, which is strictly and accurately regulated, controls the final functionality. For instance, the C subunit (PP2A-C) shows different methylation and phosphorylation sites of the C-terminal tail, which regulates the affinity for specific B subunits, thus influencing the final substrate specificity in which the enzyme exerts its function and regulates precise pathways. Ubiquitination also exerts an important role in PP2A functionality. MID1, an E3 Ligase, regulates PP2A-C ubiquitination, and together with Alpha4, it forms a trimeric complex Mid1- Alpha4-PP2A-C, which inhibits PP2A degradation [89, 90]. In addition, the proper spatial distribution of PP2A governed by its heterotrimeric organization is likely a key determinant in the modulation of downstream cellular activities [180]. PP2A has been observed to exhibit compartment-specific functions, with cytoplasmic PP2A regulating cell growth and survival [189] and mitochondrial membrane-associated PP2A promoting apoptosis [190]. By contrast, nuclear PP2A has been demonstrated to modulate chromosome stability and chromatid segregation [191, 192].

Despite these complexities, several therapeutic strategies targeting the modulation of PP2A are currently under active investigation. These approaches can be broadly categorized into (i) direct pharmacological activation or inhibition of PP2A holoenzymes, (ii) selective targeting of regulatory subunits to restore tumor-suppressive phosphatase activity, and (iii) disruption of endogenous PP2A regulators [110]. Endogenous PP2A regulators can be further classified into inhibitory, activating, and dual-function proteins. Among the inhibitors, SET directly [193] binds to the PP2A-C subunit, blocking its activity [194], while CIP2A interferes with the dephosphorylation of critical oncogenic targets, including c-MYC and Akt [195], thereby stabilizing malignant pathways. Moreover, a recent study reported that CIP2A knockdown led to cell cycle arrest in the G1 phase and enhanced cellular senescence in HCC [111]. Bortezomib also inhibits tumor growth through the induction of autophagy in HCC through the CIP2A-PP2A-AKT-4EBP1 pathway [196]. Conversely, activators, such as ceramide and PTPA, enhance PP2A function. Ceramide disrupts inhibitory complexes (e.g., PP2A-SET) [197], while PTPA binds to PME-1 to promote the catalytic reactivation of PP2A [198]. Furthermore, dual-function proteins, such as LCMT-1 and PME-1, modulate PP2A activity through methylation [199] and demethylation [94] of the C subunit, depending on the cellular microenvironment. In accordance with these strategies, a wide range of PP2A regulators is being extensively tested in preclinical (in vitro and in vivo) and clinical studies. Table 5 summarizes these agents, focusing on those targeting endogenous inhibitors and the A and C subunits of PP2A, along with their respective mechanisms of action and commonly reported side effects.

**Table 5:** Investigational drugs targeting PP2A endogenous inhibitors and PP2A subunits

Drugs	Tumor Types	Side Effects	Mechanisms and Targets
PP2A-activating drugs targeting endogenous inhibitors			
Erlotinib	HCC HNC Breast cancer Leukemia [200, 201]	Folliculitis Diarrhea [202–204]	Reduces CIP2A expression; increases p-Akt
Bortezomib	Multiple myeloma HCC [205, 206]	Peripheral neuropathy Thrombotic microangiopathy Acute interstitial nephritis [207]	Induces apoptosis in TNBC cells by downregulating CIP2A-dependent p-Akt [208]
Celastrol	Liver cancer Breast cancer Prostate cancer Multiple myeloma Glioma [209]	Infertility Cardiac cytotoxicity Hepatotoxicity Nephrotoxicity [210]	Targets CIP2A, promoting its interaction with Hsp70-interacting protein (CHIP), triggering ubiquitination and leading to subsequent CIP2A degradation [211]
TD-19	NSCLC [212]	No clinical trial	Induces apoptosis through CIP2A/PP2A/p-AKT pathway [212]
FTY720	AML [213] CLL [214]	Cardiotoxicity Lymphopenia	Inhibits SET–PP2A binding [213]; activates protein kinase C, inhibits PP2A by phosphorylation of PP2A-C T307, activates PP2A-A, and requires intact/unmutated B55α [215–218]
OSU-2 S	Mantle cell lymphoma NSCLC [219, 220]	Interactions with the immune system [221]	Inhibits the SET–PP2A binding [222]
MP07-66	CLL [216]	Under investigation	Disrupts the SET–PP2A complex [216]
TGI1002	Chronic myeloid leukemia (CML) [223]	No clinical trial	Inhibits the SET–PP2A interaction and increases PP2A activity [223]
ABL-127	Endometrial adenocarcinoma Xenograft tumor models [224]	No clinical trial	Inhibits the PME-1- PP2A-A–PP2A-B interaction [224]
PP2A-inhibiting drugs targeting specific PP2A subunits			
SMAPs	PDAC [225, 226]	No clinical trial	Binds to A subunit, inhibiting PP2A assembly with B56α [107]
LB-100	Resistant HER2-positive breast cancer CRC [227]	Adverse splicing events [228]	Binds to the C subunit and disrupts/impairs the holoenzyme conformation [229]

### PP2A inhibition as a therapeutic strategy

One of the most promising candidates for the inhibition of PP2A is LB100, a synthetic small molecule [230]. LB100 is a possible option for enhancing the effectiveness of cancer treatments, particularly in GBM therapy. Preclinical studies have demonstrated that LB-100-mediated PP2A inhibition potentiates CAR-T cell efficacy in GBM through mTORC1 pathway activation [24]. LB-100 demonstrates broad therapeutic potential extending beyond GBM to bladder cancer, in which PP2A inhibition mechanistically modulates p21 regulation. Biochemical evidence indicates that PP2A mediates the dephosphorylation of phospho-p21 at the S130 residue, thereby stabilizing p21 and promoting cell cycle arrest. By blocking this activity in a dose-dependent manner, LB-100 promotes the accumulation of phosphorylated p21, which is subsequently targeted for proteasomal degradation. This reduction in functional p21 levels facilitates cell cycle progression and enhances tumor sensitivity to cytotoxic therapies [231]. Table 6 outlines the clinical trials evaluating LB100 in cancer therapy.

**Table 6:** Clinical trials investigating LB-100 alone or in combination for cancer therapy

Study/Phase	Status	Study Overview	ClinicalTrials.gov ID
LB-100 with docetaxel in solid tumors, Phase I	Completed 2017	Assessment of LB-100 catalytic inhibition against PP2AC and PPP5C (protein phosphatase 5 catalytic subunit) [227]	NCT01837667
LB100 in recurrent GBM, Phase II	Completed 2023	Determination of LB100 pharmacokinetic properties [232]	NCT03027388
LB-100, carboplatin, etoposide, and atezolizumab in untreated extensive stage SCLC, Phase I	Active 2024	Investigation of side effects and optimal dosing in a combinatory approach [233]	NCT04560972
LB-100 and atezolizumab in metastatic stable CRC, Phase I	Recruiting 2024	Evaluation of the side effects and optimal dosage	NCT06012734
LB-100 combined with dostarlimab in ovarian clear cell carcinoma, Phase I/II	Recruiting 2024	Assessment of the effectiveness of a combinatory therapy targeting PP2A [119]	NCT06065462
LB-100 combined with doxorubicin in advanced soft tissue sarcomas, Phase I/II	Active 2025	Evaluation of combination therapy dosing (dose-finding study)	NCT05809830
LB-100 in low or intermediate-1 risk myelodysplastic syndromes (MDS), Phase I/II	Recruiting 2021	Evaluation of the safety and therapeutic potential of the drug LB-100 in treating MDS	NCT03886662

Alternative PP2A inhibitors, including okadaic acid and dasatinib, have demonstrated efficacy in chronic myeloid leukemia (CML) models. Mechanistic studies have revealed that these compounds reduce both PP2A enzymatic activity and the expression of its structural subunits. Treatment with these inhibitors induces significant biological consequences in CML cells, including (i) the activation of apoptotic pathways and (ii) the induction of cell cycle arrest [69]. In prostate cancer with *BRCA2* deficiency, the pharmacological inhibition of PP2A through PP2Ai induces synthetic lethality through the reactivation of the spindle assembly checkpoint. This targeted approach capitalizes on the inherent genomic instability of *BRCA2*-mutant cells, resulting in mitotic catastrophe and selective tumor cell elimination while preserving normal tissue function [234]. At first glance, it seems counterproductive that pharmacological PP2A inhibition can be useful for a molecule with almost exclusively tumor-suppressive effects. One can only speculate that this can always be useful for tumors and concomitant therapies, in which the primary goal is to subject tumor cells to proliferation stress and thus drive them into apoptosis.

### PP2A activation and restoration as a therapeutic strategy

Conversely, FTY720, also known as fingolimod, serves as an activator of PP2A designed to restore its function in cancer types in which PP2A activity is suppressed [235, 236]. By reactivating PP2A, FTY720 has the potential to counteract tumor growth in malignancies [113, 235] such as GBM [237]. Moreover, FTY720 acts as an immunosuppressant and is applied in the treatment of multiple sclerosis and cancer research [235, 238]. For instance, FTY720 activated PP2A in multiple myeloma, leading to the dephosphorylation of the AMP-activated protein kinase subunit α. This resulted in the reduced expression of phosphorylated eEF2, consequently triggering myeloma cell death [235]. The molecule DT-061 serves as a further activator of PP2A, which stabilizes the fully assembled B56α-PP2A holoenzyme, thus keeping it in an active state. This stabilization mechanism offers therapeutic potential through sustained phosphatase activity against key oncogenic targets, most notably the transcription factor c-MYC, which plays a pivotal role in melanoma pathogenesis [107, 114]. In KRAS-mutant lung cancer, DT-061 demonstrates synergistic activity when combined with the MEK inhibitor AZD6244. The combination therapy promotes the PP2A-mediated suppression of oncogenic signaling through the concurrent inhibition of p-Akt and MYC, ultimately leading to tumor regression in preclinical models. These findings position DT-061 as a promising therapeutic candidate for both MYC- and KRAS-driven malignancies, with mechanistic versatility across different cancer types [239]. Similar studies using PP2A activators in GBM have reported two reagents, namely, NZ-8–061 and DBK-1154, which effectively cross the blood–brain barrier and exhibit potent antitumor activity. Notably, the oral administration of DBK-1154 in mouse models resulted in a substantial reduction in intracranial tumor growth and significantly increased survival rates [240]. In many cancers, particularly those driven by hyperactive kinase signaling, such as NSCLC [241], SOC [242], AML [102], and breast cancer [6], PP2A predominantly functions as a tumor suppressor. In such circumstances, the reactivation of PP2A restores its physiological function as a regulatory mechanism that controls oncogenic signals. Furthermore, studies have demonstrated that the restoration of PP2A activity can enhance the sensitivity of tumors to chemotherapeutic agents and kinase inhibitors, particularly in cases of drug-resistant models [103]. As PP2A can also have tumorigenic effects, it could be useful to inhibit PP2A in certain contexts and in certain tumor tissues. The dephosphorylation of c-MYC at T58 by PP2A-B56α [187] and at S62 by PP2A-B55α [188] represents a critical regulatory switch, with the former promoting tumor-suppressive degradation and the latter enabling tumorigenic stabilization and transcriptional activation. Similarly, in Wnt/β-catenin signaling, specific PP2A complexes dephosphorylate β-catenin at residues required for degradation, facilitating its nuclear accumulation and the activation of growth-promoting transcriptional programs [185]. In such tumors, PP2A inhibition may suppress oncogenic transcription and limit proliferation. PP2A can also sustain survival signaling in hematological malignancies and contribute to resistance against pro-apoptotic therapies [113]. The inhibition of PP2A in these contexts may lower the apoptotic threshold. Moreover, in the immune system, PP2A negatively regulates T-cell activation and persistence. Transient PP2A inhibition in T cells has been shown to enhance antitumor immunity and improve the efficacy of CAR-T cell therapies [24]. A comprehensive understanding of these functional distinctions in various cancers is vital for developing innovative modalities that harness their dual functionality in oncology.

### Fine-tuned regulation through subunit-specific expression

Holoenzymes achieve precise biological regulation through their modular structure, in which variable regulatory subunits control substrate specificity, localization, and activity. This structural plasticity allows a single catalytic core to perform diverse functions, depending on its associated partners. In addition, the presence of splice variants and protein isoforms confers a further range of variability in enzyme assembly. In cancer, malignant cells frequently dysregulate this system by altering subunit expression patterns, creating pro-tumorigenic holoenzyme configurations that drive oncogenic signaling and confer therapeutic resistance [243]. Over the past few decades, growing scientific evidence has highlighted the critical roles of specific PP2A subunits, shedding light on their unique regulatory features. Given the dual role of PP2A in malignancy, it may be useful to address individual subunits. An interesting example of this is B55γ, which enables important novel therapeutic possibilities, even if the functional significance of B55γ/*PPP2R2C*, including its transcriptional variants and protein isoforms, is incompletely understood. B55γ/*PPP2R2C* containing holoenzymes has been observed to play critical roles in DNA repair, apoptosis regulation, and metabolic reprograming through its interactions with key signaling proteins. In DNA repair, B55γ-containing holoenzymes might be able to regulate the DNA damage response by interacting with SIK2, which has been observed to exert a regulatory function in DNA damage response [244]. In apoptosis regulation, it has been observed that B55γ serves a regulatory function in response to glucose starvation [108]. Thus, we focus on *PPP2R2C*, synthesizing the most recent advances.

### Effect of *PPP2R2C* expression on therapy resistance and PP2A modulation

Indisputably, one of the most persistent challenges in modern oncology is therapy resistance, a phenomenon in which cancer cells evolve mechanisms to evade treatments, leading to disease recurrence and poor outcomes. This resistance is often driven by genetic and epigenetic alterations, TME interactions, and cellular plasticity [245]. Among the most aggressive and treatment-resistant cancers is GBM, a high-grade brain tumor notorious for its resilience to conventional therapies, including chemotherapy and radiation. The complexity of treating GBM is further compounded by the blood–brain barrier, which restricts the delivery of therapeutic agents to the brain, and the tumor’s remarkable heterogeneity. GBM exhibits intertumoral (differences between patients) and intratumoral (differences within the same tumor) heterogeneity, which contributes to its adaptability and resistance. This heterogeneity is driven by diverse genetic mutations, clonal evolution, and the presence of cancer stem cells (CSCs), which are particularly adept at surviving therapy and driving tumor recurrence [246]. In this context, *PPP2R2C* is of particular importance because of its almost exclusive occurrence in the brain [10] and its contribution primarily to brain disorders (neurodegenerative diseases, learning disabilities, and brain tumors). Its expression is highly tissue specific, with elevated levels predominantly observed in neuronal tissues, such as the cerebral cortex and basal ganglia, which enables some degree of specificity as a tumor target and provides further proof of its key role in neuronal cells [247]. The reduced expression of this subunit has been consistently reported in GBM in a stage-dependent manner [10]. Further investigations have demonstrated that this downregulation improves glucose homeostasis (favoring gluconeogenesis) by suppressing S6K phosphorylation. Mechanistically, B55γ stabilizes SIK2 through direct interaction, and this B55γ–SIK2 axis is essential for inhibiting S6K phosphorylation in glioma cells [108]. The dysregulation of glucose metabolism is one of the main targets of GMB and other cancers, which base their survival mainly on glycolysis as their primary method of energy derivation, a process known as the Warburg effect [248]. Therefore, metabolic reprograming plays a pivotal role in driving cancer treatment resistance. Under therapeutic pressure, tumor cells frequently rewire their metabolic networks, diminishing their therapeutic efficacy. Exploiting these adaptive mechanisms is a promising strategy for enhancing treatment outcomes.

Moreover, reduced PP2A activity due to PPP2R2C dysfunction hyperactivates survival pathways, such as PI3K/AKT and MAPK/ERK, further enabling cancer cells to evade apoptosis and enhance survival therapeutic stress [249]. Accordingly, *PPP2R2C* has been proposed to act as a tumor suppressor in brain cancer and as a biomarker for tumor progression and prognosis. Consistent with this finding in GBM, the loss of the *PPP2R2C* subunit was associated with an increase in prostate cancer cell proliferation, particularly in resistant cases to androgen ligand depletion, suggesting that *PPP2R2C* plays a suppressive role in GBM and prostate carcinogenesis, indicating its potential as a therapeutic target in the management of these diseases [10]. This and another study indicated that PPP2R2C is an effective suppressor of the mTOR/S6K pathway [109]. In addition, recent studies have identified PP2A as a negative regulator of CD8^+^ T-cell effector functions in GBM, in which it suppresses T-cell receptor (TCR) signaling by dephosphorylating key activation nodes [250]. As a major goal of immunotherapy is to enable CD8^+^ T-cells to attack tumors with the same efficiency as pathogen-infected cells, the role of PP2A in dampening TCR and cytokine responses presents a critical barrier. Notably, enrichment of the PP2A regulatory subunit *PPP2R2C*, together with other phosphatases, has been observed in PyMT tumor-infiltrating CD8^+^ T-cells, suggesting a broader mechanism of phosphatase-mediated immunosuppression [251]. This has direct implications for CAR-T cell therapy, as PP2A activity may limit cytotoxic efficacy, highlighting its potential as a therapeutic target to enhance immune responses [24]. In summary, PP2A has both tumorigenic and tumor-suppressive properties. Therefore, it is important to describe exactly which of the many regulatory subunits is involved in which cellular context. The situation is also further complicated by the fact that there are different splice variants of almost all regulatory subunits whose functions are unclear. This knowledge must be further evaluated in the context of both the pathological examination of cancer tissues and liquid biopsies. Such insights are crucial not only for the targeting of PP2A subunits and/or transcript variants in pathological evaluations but also for their potential use as tumor markers in liquid biopsy applications.

### Role of *PPP2R2C* splice variants in resistance

In eukaryotic cells, alternative splicing allows a small number of genes to produce a wide variety of proteins. While the mechanisms and effects of splicing in single transcripts are fairly well known, recent research has shifted the focus to understanding splicing networks. Studies have revealed that these networks work in a coordinated manner to control tissue and organ development, playing a crucial role in various human developmental processes [252]. This process becomes dysregulated in malignancies and contributes to multiple cancer hallmarks [193]. Remarkably, a large number of human protein-coding genes (about 75%) produce multiple isoforms [253], creating significant challenges for precision medicine. A comprehensive drug–target analysis revealed that 76% of 883 small-molecule oncology therapeutics could either miss their intended isoform or affect unintended variants in healthy tissues [254]. This molecular heterogeneity provides tumors with an evolutionary advantage. Individual cancer cells can express different isoform profiles [255], creating a reservoir of potential resistance mechanisms under therapeutic pressure. Clinically relevant examples include *HER2* splice variants (lacking exon 16) that confer resistance to trastuzumab [256]. Similar escape mechanisms have been documented in advanced immunotherapies, particularly CAR-T treatments targeting EGFRvIII in GBM or CD19 in B-cell leukemia [257]. In addition, splicing variants of genes such as *p53* [258], *BRCA1* [259], and *MET* [260] have been shown to alter cellular functions in ways that either promote or suppress cancer.

Unfortunately, due to the elusive nature of PP2A’s mechanisms and the limited research on *PPP2R2C* splice variants, there is a significant gap in understanding their role in cancer resistance. Currently, there is little direct evidence connecting specific *PPP2R2C* splice variants to treatment-resistant GBM or prostate cancer. While the PP2A complex (containing *PPP2R2C*) is known to influence cancer progression, major genomic studies, such as TCGA and CPTAC, have not systematically examined the gene’s alternative splicing patterns. Understanding how these variants influence PP2A’s tumor-suppressive functions could reveal novel therapeutic targets and strategies to overcome cancer resistance, ultimately advancing precision oncology.

### *PPP2R2C* as a predictive biomarker

A predictive tumor marker is a biomarker that can (in some cases) be used to indicate the presence of cancer or, more often, to characterize the tumor and its responsiveness to therapy. Biomarkers play a critical role in oncology, in which treatment responses vary significantly across cancer types and even among patients with the same cancer. While some individuals may benefit dramatically from a given treatment, others may experience only its side effects without therapeutic gain [261]. This variability underscores the importance of predictive testing to guide personalized treatment decisions, ensuring that patients receive therapies that are most likely to help them while avoiding unnecessary toxicity. The integration of predictive biomarkers into clinical practice represents a cornerstone of precision oncology, allowing clinicians to match patients with optimal treatments while avoiding ineffective therapies.

In recent years, the expression of *PPP2R2C*, which encodes for the B55γ protein, has gained increasing attention due to its unique, tissue-specific distribution and emerging role in tumor biology. Its restricted expression pattern suggests a critical function in maintaining PP2A’s substrate specificity and cellular homeostasis, with dysregulation contributing to oncogenic transformation. Moreover, aberrant expression alongside mutation and deletion of PP2A has been commonly observed across several cancer types, such as prostate, breast, AML, and primary plasma leukemia [77, 262, 263]. For instance, *PPP2R2C* missense mutation of R274H/C/S at the exon 7 level has been reported in multiple malignancies, as reported in cBioPortal (Supplementary Fig. 1). Therefore, *PPP2R2C2* shows peculiar characteristics that suggest its potential as a predictive biomarker in specific cancer types. Notably, the PP2A-B55 phosphatase complex plays a pivotal role in cell cycle control, with the B55γ subunit (encoded by *PPP2R2C*) emerging as a linchpin in developmental and oncogenic pathways. Recent studies have highlighted B55γ’s dual role as both a mitotic safeguard and a key determinant of osteoblast maturation, underscoring its tissue-specific regulatory functions [264]. Thus, the dysregulation of this subunit could disrupt lineage commitment in bone formation while simultaneously driving mitotic errors in cancer, a duality that warrants further exploration for targeted therapies [264]. In this regard, the telomere position effect (TPE) and TPE-Over Long Distance (TPE-OLD) may also regulate the expression of PP2A subunits, influencing the holoenzyme final assembly and therefore the substrate affinity, which modulates the activation or inhibition of specific pathways. For instance, *PPP2R2C* has already been characterized as a TPE-OLD gene, showing a specific expression inversely correlated with telomere length [109]. The bioinformatics approach highlighted the possibility that other PP2A subunits could also be modulated by telomere length and positioning, providing further insights into how this mechanism fine-tunes cellular processes during aging and malignancies (Supplementary Fig. 2) [265]. Notably, most malignancies aim to maintain a sufficient length of telomeres in order to enhance their duplication rate and survival [266]. In addition, it has been demonstrated that PP2A directly affects human telomerase reverse transcriptase activity [267]. Taking all the above mentioned hallmarks into account, it is undeniable that further investigation of PP2A subunits could lead to improvements in precision medicine and support their potential use as preventive biomarkers.

### TME effects

TME plays a pivotal role in cancer progression and therapeutic strategies, shielding cancer cells from the host’s immune system through the expression of cytokines and chemokines that regulate cell growth, survival, and metastasis [268]. Cancer cells typically exhibit a highly environment-dependent survival niche, which means that they rely heavily on specific conditions within their microenvironment to thrive. Outside this niche, they often undergo spontaneous cell death due to the activation of apoptotic pathways, for example, in chronic lymphoblastic leukemia [269]. Therefore, cancer cells are prone to modifying their location environment conditions to promote pH imbalance or a hypoxic state that supports their survival [270, 271]. In this context, various serine/threonine and tyrosine protein phosphatases control the signaling pathways that directly influence those processes. PP2A exerts an essential function in regulating hypoxia signaling pathways. For example, in GMB, a hypoxic environment enhances PP2A activity mediated by the HIF1α pathway, supporting the survival of GBM-CSCs. Recent studies have suggested that PP2A-mediated cell cycle arrest in GBM-CSCs occurs through the putative dephosphorylation of polo-like kinase 1, while the concomitant activation of DNA repair mechanisms promotes CSC survival during growth arrest [272]. Although hypoxia-induced suppression of the mammalian target of rapamycin (mTOR) substrate phosphorylation occurs independently of PP2A in HEK293 cells [273], this transformed embryonic kidney cell line may not fully recapitulate PP2A’s functional significance in neoplastic cells. Emerging evidence suggests that the HIF1α–PP2A axis mediates the cross-talk between mTOR and ERK pathways in hypoxic ovarian cancer microenvironments [274].

In this review, we highlight several examples of how PP2A influences TME. The roles of PP2A and other protein phosphatases in the context of cancer microenvironments were thoroughly reviewed by Ruvolo in 2019 [275].

#### Therapeutic complications and off-target effects

Developing PP2A modulators as therapeutic agents presents several significant challenges that could be categorized into therapeutic complications, off-target effects, and issues related to the lack of isoform selectivity. Due to its pleiotropic functions, the modulation of PP2A activity can have widespread and sometimes unpredictable effects on cellular homeostasis, leading to unintended consequences such as impaired immune responses or metabolic dysregulation [276]. For instance, when PP2A is inhibited or mutated, particularly in its structural subunit PP2A-Aα (e.g., E64D, R418W mutations) or regulatory subunits, such as B55α/B56γ, cells lose critical control over survival and proliferation signals [137, 277]. This results in the sustained activation of kinases, such as Akt and MAPK, driving tumor growth in melanoma, GBM, and CRC [114, 278–280]. Conversely, in neurodegenerative diseases, excessive PP2A activity can be harmful. Overactive PP2A hyperdephosphorylates tau and other synaptic proteins, contributing to Alzheimer’s neurofibrillary tangles and autism-related synaptic pruning defects [281]. Isoform-specific dysregulation further complicates the picture. For instance, suppressed B55α disrupts cell–cycle proteins such as Cyclin D1, promoting metastasis in breast and lung cancers [282], while overexpressed B56γ paradoxically inactivates PP2A in T-cell leukemia [283]. Notably, several studies have demonstrated that PP2A is involved in CD4^+^ T-cell activation and differentiation [284, 285]. This activation is achieved through the dephosphorylation of IκB kinase (IKK) within the TCR signaling cascade. By modulating IKK activity, PP2A facilitates the nuclear translocation of NF-κB, driving toward the transcriptional upregulation of key inflammatory mediators, such as IL-2 and IFN gamma [286]. In this scenario, endogenous regulators of PP2A play a leading role in its activity modulation.

Pathogens also exploit PP2A. Viral proteins, such as SV40 small t antigen, hijack PP2A-Aα to dysregulate Wnt signaling [287], and the oncoprotein CIP2A blocks PP2A-B56α to stabilize c-MYC in HNC [195]. Therapeutically, strategies vary by context. Restoring PP2A activity (e.g., with FTY720 in leukemia) can counteract cancer, while transient PP2A inhibition (e.g., LB100 in GBM) may sensitize tumors to therapy [180]. These opposing approaches highlight the need for precision targeting based on PP2A’s role in each disease.

## Conclusion and future perspectives

The multiple facets of PP2A confer a particular interest to this holoenzyme in cancer therapies and dysfunctional brain development disorders. Hundreds of possible different conformations provide specific affinity to substrates, in which phosphorylation is essential for the activation of signaling cascades, leading to the regulation of key pathways. In this review, we summarize the common dysregulation of PP2A in different cancers and the latest clinical approaches, providing a special focus on the role of B55γ, an underestimated regulatory subunit that has recently shown triggering aspects due to its specific tissue expression. Novel cancer therapies are necessary to cross the resistance of tumors sheltered by their specific microenvironments, enhancing the importance of studying personalized therapies.

Nevertheless, newer research that highlights specificity and delivery efficiency remains a major obstacle when targeting splicing events or abnormal RNA species at the post-transcriptional level.

Therefore, future studies on regulatory subunits and their variants are essential to understand their roles and their potential as predictive biomarkers, which are extremely important for predicting adverse clinical outcomes.

## Supplementary Information

Supplementary Material 1: Fig. S1. Mutation frequency of PPP2R2C in multiple malignancy reported in cBioPortal. (Access: 20 May 2025). Fig. S2. TPE-OLD candidates of PP2A subunits which expression is regulated by telomere length. https://tpe-old.uni-rostock.de/ (Access: 20 June 2025).

## Data Availability

No datasets were generated or analysed during the current study.
