# Supplementary material for: Targeting PP2A in cancer: an underrated option
Source: J Exp Clin Cancer Res. 2025 Oct 27;44:295. doi: 10.1186/s13046-025-03560-y (PMC12557939; doi:10.1186/s13046-025-03560-y)
Supplement: Supplementary file 1 — Supplementary Material 1: Fig. S1. Mutation frequency of PPP2R2C in multiple malignancy reported in cBioPortal. (Access: 20 May 2025). Fig. S2. TPE-OLD candidates of PP2A subunits which expression is regulated by telomere length. https://tpe-old.uni-rostock.de/ (Access: 20 June 2025). [file 13046_2025_3560_MOESM1_ESM.docx]

**Supplementary Figures**

**
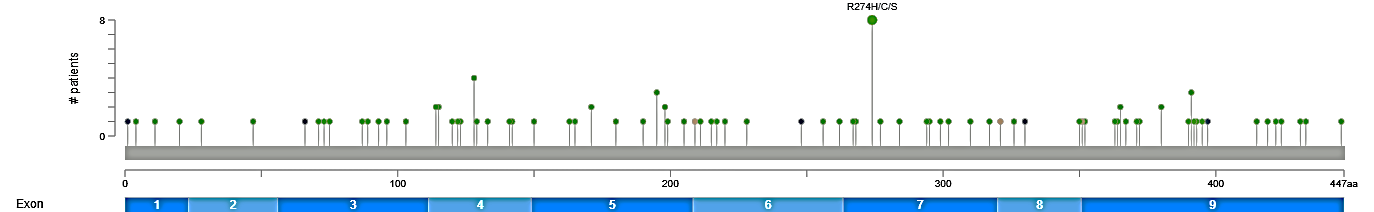
**

**Supp. figure 1. Mutation frequency of PPP2R2C in multiple malignancy reported in cBioPortal. (Access: 20 May 2025)**


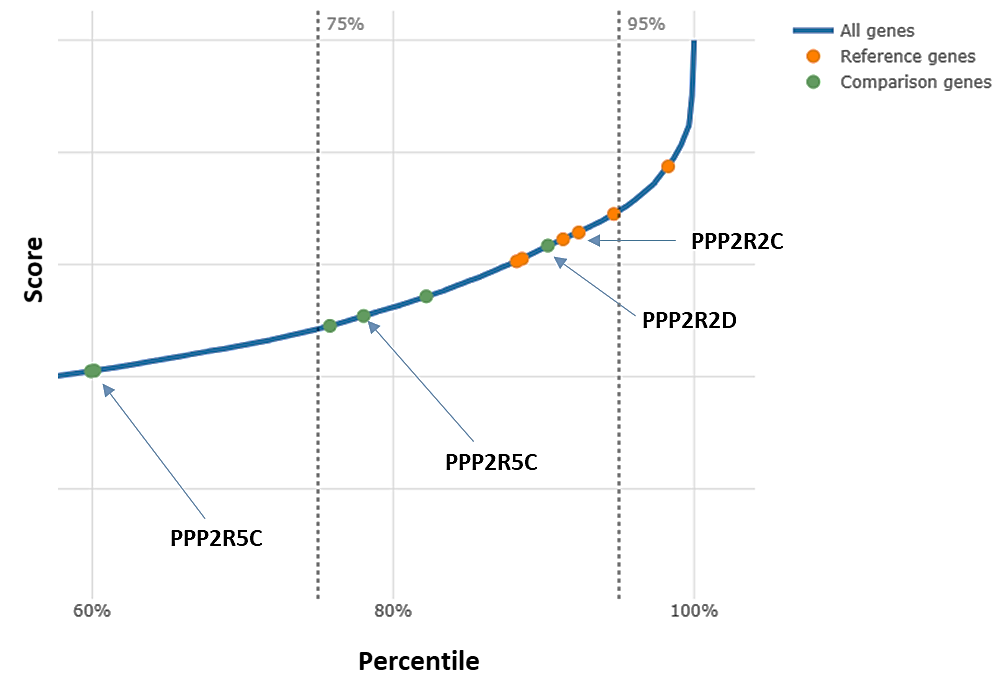


**Supp. figure 2. TPE-OLD candidates of PP2A subunits which expression is regulated by telomere length.**

**https://tpe-old.uni-rostock.de/ (Access: 20 June 2025)**
